# Advancing a Hybrid Decision-Making Model in Anesthesiology: Applications of Artificial Intelligence in the Perioperative Setting

**DOI:** 10.3390/healthcare14010097

**Published:** 2025-12-31

**Authors:** Gilberto Duarte-Medrano, Natalia Nuño-Lámbarri, Daniele Salvatore Paternò, Luigi La Via, Simona Tutino, Guillermo Dominguez-Cherit, Massimiliano Sorbello

**Affiliations:** 1 Anesthesiology Department of Hospital Medica Sur, Mexico City 11510, Mexico; dr.gilbertoduartem@gmail.com (G.D.-M.); g.dominguez.cherit@gmail.com (G.D.-C.); 2 Translational Research Unit, Medica Sur Clinic & Foundation, Mexico City 11510, Mexico; nlambarri@gmail.com; 3 Department of Surgery, Faculty of Medicine, The National Autonomous University of Mexico (UNAM), Mexico City 14050, Mexico; 4 Department of Anesthesia and Intensive Care, Hospital “Giovanni Paolo II”, ASP Ragusa, 97100 Ragusa, Italy; paternomd@icloud.com (D.S.P.); simona.tutino.29.9.92@gmail.com (S.T.); massimiliano.sorbello@unikore.it (M.S.); 5 Department of General Surgery and Medical Surgical Specialties, University of Catania, 95123 Catania, Italy; 6 Faculty of Medicine and Surgery, University of Enna “Kore”, 94100 Enna, Italy

**Keywords:** artificial intelligence, machine learning, anesthesiology, perioperative care, clinical decision support

## Abstract

Artificial intelligence (AI) is rapidly transforming anesthesiology practice across perioperative settings. This review explores the evolution and implementation of hybrid decision-making models that integrate AI capabilities with human clinical expertise. From historical foundations to current applications, we examine how machine learning algorithms, deep learning networks, and big data analytics are enhancing anesthetic care. Key applications include perioperative risk prediction, AI-assisted patient education, automated analysis of clinical records, airway management support, predictive hemodynamic monitoring, closed-loop anesthetic delivery systems, and pain management optimization. In procedural contexts, AI demonstrates promising utility in regional anesthesia through anatomical structure identification and needle navigation, monitoring anesthetic depth via EEG analysis, and improving quality control in endoscopic sedation. Educational applications include intelligent simulators for procedural training and academic productivity tools. Despite significant advances, implementation challenges persist, including algorithmic bias, data security concerns, clinical validation requirements, and ethical considerations regarding AI-generated content. The optimal integration model emphasizes a complementary approach where AI augments rather than replaces clinical judgment—combining computational efficiency with the irreplaceable contextual understanding and ethical reasoning of the anesthesiologist. This hybrid paradigm reinforces the anesthesiologist’s leadership role in perioperative care while enhancing safety, precision, and efficiency through technological innovation. As AI integration advances, continued emphasis on algorithmic transparency, rigorous clinical validation, and human oversight remains essential to ensure that these technologies enhance rather than compromise patient-centered anesthetic care.

## 1. Introduction

The development of artificial intelligence (AI) has traversed a remarkable journey since its conceptual origins in the 19th century. Charles Babbage and Ada Lovelace established the foundation for modern computing by designing the “Analytical Engine,” widely regarded as the precursor to programmable computers. However, the pivotal turning point emerged from Alan Turing’s seminal work in 1950, when he introduced the renowned “Turing Test” as a criterion for evaluating whether a machine could exhibit human-like intelligence [1]. The term “artificial intelligence” was formally coined during the 1956 Dartmouth Conference, marking the establishment of a discipline dedicated to emulating human cognitive processes through machines. The 1960s and 1970s witnessed the emergence of pioneering medical expert systems, such as MYCIN and INTERNIST-1, which supported clinical decision-making through knowledge-based rules and inference engines [2,3]. The subsequent decades saw the development of increasingly sophisticated diagnostic tools like DXplain, which facilitated differential diagnosis through probabilistic reasoning. By the late 20th and early 21st centuries, the paradigm shifted toward machine learning approaches, substantially enhancing the predictive and analytical capabilities of automated systems, particularly in medical imaging and clinical diagnostics [4]. The past decade has been characterized by the profound impact of deep learning methodologies, enabling the creation of complex models capable of competing with human specialists, most notably in dermatology, radiology, and natural language processing. The integration of AI into technologies such as IBM’s Watson, virtual clinical assistants, and automated detection systems has solidified its role in personalized medicine, surgical planning, and perioperative care, revolutionizing numerous aspects of healthcare delivery and research. AI refers to the capacity of computer systems to perform tasks that traditionally require human intelligence, including reasoning, decision-making, and pattern recognition. In healthcare, AI is increasingly utilized to support diagnostic accuracy, predict clinical outcomes, tailor therapeutic interventions, and optimize operational workflows. In anesthesiology, AI-enabled decision support systems have been explored for intraoperative hemodynamic monitoring, early prediction of hypotension, and automated titration of anesthetic agents. Machine Learning (ML), a subfield of AI, focuses on the development of algorithms that enable systems to learn from data and improve performance over time without being explicitly programmed. ML algorithms such as linear regression, random forests, and k-nearest neighbors are employed in both supervised and unsupervised learning contexts. In perioperative medicine, ML has been applied to predict postoperative complications, stratify patient risk, and assist in resource allocation based on preoperative variables [5]. Deep Learning (DL) is an advanced subset of ML that leverages artificial neural networks with multiple layers to identify complex, non-linear patterns in large datasets. Convolutional neural networks (CNNs), for instance, have demonstrated high performance in analyzing radiological and ultrasound images, including point-of-care ultrasound (POCUS) in airway assessment or echocardiographic interpretation for anesthetic management in high-risk surgical patients [6].

Big Data refers to extremely large and heterogeneous datasets that surpass the analytical capabilities of traditional data processing tools. In medicine, Big Data encompasses genomic sequences, electronic health records, perioperative physiological monitoring data, and imaging repositories. The integration and analysis of such datasets through AI-driven methods facilitate precision medicine, enabling individualized perioperative care, prediction of adverse events, and real-time clinical decision support systems in anesthesiology [7].

## 2. Materials and Methods

A structured literature search was conducted between January 2020 and May 2025, using the electronic databases PubMed, Scopus, and Web of Science. The search focused on peer-reviewed English-language publications reporting the development, validation, or clinical evaluation of AI systems relevant to anesthetic practice. No geographic restrictions were applied. The following search terms and Boolean combinations were used: “artificial intelligence” AND “anesthesiology” “machine learning” AND “perioperative care” “deep learning” AND “airway management” “closed-loop anesthesia” OR “decision support systems” “hemodynamic prediction” OR “Hypotension Prediction Index (HPI)” “large language models” OR “chatGPT” AND “medical communication” OR “education” “AI” AND “analgesia”, “sedation”, “PONV prediction”. We included studies fulfilling the following inclusion criteria: Published in English between 2020 and 2025, Original clinical research (RCTs, cohort studies, observational studies), systematic reviews, or meta-analyses directly involving AI applications within anesthesiology or perioperative medicine, reporting clinical outcomes, predictive performance (e.g., AUROC), or implementation feasibility. The initial search yielded 268 articles. After removing duplicates and screening titles and abstracts for relevance, 71 full-text articles were evaluated. Based on the criteria above, 35 studies were ultimately included in this review. Additional sources were identified through backward citation tracking of key publications.

## 3. Clinical Applications of Artificial Intelligence in Anesthesiology

### 3.1. Perioperative Risk Prediction: ASA Classification and Postoperative Complications

AI has demonstrated significant efficacy in automated perioperative risk stratification, including the prediction of adverse events and ASA classification. ML models have been successfully trained to anticipate post-induction hypotension, a common complication that increases the risk of myocardial injury, delirium, and prolonged hospital stays. In a comprehensive analysis of 5406 patients, Chen et al. evaluated various algorithms and found that logistic regression models achieved an AUROC of 0.74, outperforming more complex models such as XGBoost and neural networks [8]. Complementary research by Çelik et al. [9] evaluated the application of generative AI models, including ChatGPT, Copilot, and Gemini, for anesthetic method selection in orthopedic patients. Their findings revealed that Gemini’s recommendations aligned with human anesthesiologists’ decisions in 68.5% of cases overall, with concordance increasing to 85.7% in polypharmacy patients. These results suggest significant potential for AI as a clinical decision support tool during preoperative assessment [9].

### 3.2. AI-Assisted Patient Education in Anesthesiology

The integration of AI into perioperative education is emerging as a valuable tool for enhancing patient-centered care, particularly in mitigating preoperative anxiety. In a randomized controlled trial, Yahagi et al. [10] evaluated the impact of an AI chatbot (ChatGPT-3.5) compared to conventional anesthesia nurse education in adult patients scheduled for surgery under general anesthesia. The intervention group, which engaged with ChatGPT to address anesthesia-related concerns, exhibited a significant reduction in anxiety levels measured by the State-Trait Anxiety Inventory at multiple time points. Notably, while no overall difference in anxiety scores between groups was observed, a significant group-by-time interaction effect was detected, suggesting that the chatbot contributed to a more dynamic reduction in anxiety over time (*p* = 0.015) [10]. This study highlights the growing role of AI in patient education within anesthesiology, not as a replacement but as a complementary agent to human care. ChatGPT’s capacity to deliver tailored, interactive, and accessible information enabled patients to express concerns in their own terms, fostering psychological comfort and engagement. Despite occasional inaccuracies, the findings demonstrate that AI chatbots can contribute meaningfully to perioperative communication strategies by reducing cognitive uncertainty and enhancing the patient’s perceived control—factors closely linked to reduced anxiety and improved postoperative outcomes.

### 3.3. Automated Analysis of Clinical Records and Medical Imaging

AI models demonstrate remarkable capability in analyzing large volumes of structured and unstructured clinical text, including medical histories, laboratory examinations, and surgical risk factors. While clinical implementation remains in its early stages, emerging research has begun exploring natural language processing for automated evaluation of clinical histories and prediction of perioperative complications [11]. In the domain of medical imaging, particularly in obstetric anesthesia, AI has been applied to automated analysis of spinal ultrasound to facilitate neuraxial block placement. Additionally, predictive models based on anatomical characteristics have been developed to forecast adequate spinal block achievement [12]. These applications represent promising advancements in precision anesthesia administration through image recognition and computational analysis.

### 3.4. Artificial Intelligence in Airway Management

Airway management represents a critical domain where artificial intelligence applications are increasingly demonstrating significant potential. AI systems are being developed to enhance prediction of difficult airways, optimize device selection, and provide real-time guidance during intubation procedures. In fact, AI can support clinical decision-making across the airway management continuum [13]. Machine learning algorithms have shown promising results in predicting difficult laryngoscopy and intubation based on preoperative assessments, with some models achieving accuracy rates exceeding 85% [14]. Specific machine learning programs applied to preoperative imaging can identify anatomical landmarks, predict the difficulty of intubation, and suggest optimal intubation strategies. In ultrasound-guided techniques, AI algorithms may assist in identifying the cricothyroid membrane with greater precision, potentially improving emergency surgical airway procedures [15]. Additionally, AI-enhanced simulation platforms are revolutionizing education by creating adaptive training scenarios that respond dynamically to learners’ actions [16]. Moreover, AI-driven software might provide real-time assistance to endotracheal intubation through videolaryngoscopy, thus serving both for learning purposes and for aid during difficult scenarios. Despite these advancements, implementation challenges remain, including concerns about over-reliance on technology, the “black box” nature of some algorithms, and the need to maintain clinical skills. The optimal approach appears to be a hybrid model where AI augments rather than replaces clinical expertise, enhancing decision-making while preserving the irreplaceable human elements of airway assessment and management [13].

### 3.5. Predictive Models of Hemodynamic Instability

Predictive intraoperative monitoring represents one of the most significant AI developments in anesthesiology. Systems such as the Hypotension Prediction Index (HPI) analyze real-time physiological signals to predict hypotension up to 15 min in advance. Shimada et al. [11] noted that while randomized controlled trials evaluating HPI remain limited with small sample sizes, its implementation demonstrated a trend toward reducing hypotensive burden, although this did not reach statistical significance in meta-analysis. These early findings suggest potential clinical utility despite the need for larger validation studies. Other authors like Wijnberge et al. [17] investigated the impact of a machine learning–based early warning system—the HPI—on intraoperative blood pressure stability in patients undergoing elective noncardiac surgery. Sixty adult patients were randomized to receive either standard care or HPI-assisted hemodynamic monitoring using the Edwards Acumen platform. The algorithm provided real-time alerts when the predicted risk of hypotension exceeded 85%, prompting clinicians to follow a structured diagnostic guidance protocol to identify and treat the underlying cause of instability. The intervention group exhibited a significantly lower time-weighted average of hypotension (0.10 mm Hg vs. 0.44 mm Hg; *p* = 0.001) and shorter cumulative duration of hypotensive episodes (median 8.0 vs. 32.7 min; *p* < 0.001), without increasing the incidence of hypertension or vasopressor use. These results suggest that AI-driven predictive systems, when coupled with structured treatment protocols, can effectively reduce the burden of intraoperative hypotension and may enhance hemodynamic precision in anesthesia practice [17].

### 3.6. Closed-Loop Systems for Drug Administration (TIVA/TCI)

AI-based closed-loop systems, particularly those applied to propofol and remifentanil administration in Total Intravenous Anesthesia (TIVA) or Target-Controlled Infusion (TCI) protocols, have demonstrated substantial improvements in predicting and controlling anesthetic depth. Wang et al. [18] developed an innovative hybrid model combining long short-term memory networks, Transformer architecture, and Kolmogorov–Arnold Networks, achieving a mean squared error of 0.0062, significantly outperforming traditional regression models. This precision enables more stable anesthetic maintenance and potentially reduces recovery time and complications associated with anesthetic depth fluctuations.

### 3.7. Algorithms for Real-Time Adverse Event Detection

AI also enables early detection of intraoperative adverse events, including patient movement, bradycardia, and hypoxemia. Furthermore, specialized models have been developed to predict complications such as postoperative nausea and vomiting. Glebov et al. [19] implemented an ensemble learning approach to predict both early and late postoperative nausea and vomiting, achieving predictive performance of 83.6% and 74.8%, respectively, substantially outperforming traditional assessment tools like the Apfel or Koivuranta scales. These advances in real-time monitoring represent a paradigm shift toward anticipatory rather than reactive anesthetic management, potentially improving patient safety and reducing postoperative complications.

## 4. Pain Management and Analgesia

AI offers significant potential to enhance the personalization of multimodal analgesia strategies. By integrating preoperative risk factors, intraoperative variables, and pharmacogenetic data, AI models can tailor analgesic regimens to individual patients. These models can optimize opioid and non-opioid administration schedules and support dynamic analgesic decision-making based on physiological and behavioral feedback. For instance, Kong et al. describe applications of AI in obstetric anesthesia where predictive models were used to optimize spinal anesthesia dosing, demonstrating a framework that could be extended to multimodal analgesia in broader populations. The models used penalized regression techniques (e.g., LASSO) to account for patient-specific anatomical and physiological factors and showed high predictive accuracy in dose determination, an approach readily applicable to analgesic titration [12]. Moreover, AI-based systems have been used to predict individual responses to opioids and other analgesics, offering early identification of patients at risk for inadequate analgesia or opioid-related adverse effects. These approaches contribute to the evolution of precision analgesia by enabling clinicians to balance efficacy and safety in real time, especially in enhanced recovery protocols.

### 4.1. Prediction of Chronic Postoperative Pain

The transition from acute to chronic postoperative pain is influenced by multifactorial risk factors, including surgical type, psychological status, and pre-existing pain syndromes. Traditional clinical models for predicting chronic postoperative pain have shown limited sensitivity. ML techniques, however, allow the integration of complex and non-linear datasets to better identify patients at high risk for persistent postsurgical pain. Although direct chronic postoperative pain prediction models were not the central focus in the reviewed articles, the studies by Glebov et al. [19] and Chen & Zhang [8] demonstrate the effectiveness of ML in perioperative outcome prediction. Glebov et al. [19] successfully used ensemble ML models to predict early and delayed postoperative nausea and vomiting, a frequent correlate of poor postoperative analgesia, achieving superior performance over conventional scoring systems. This framework is transferable to chronic pain prediction by incorporating psychosocial metrics, opioid exposure data, and intraoperative nociception monitoring. Furthermore, real-time prediction of post-induction hypotension, as explored by Chen & Zhang [8], illustrates the capacity of ML models to anticipate complex perioperative events using routinely collected data—a methodology that can also be applied to long-term pain outcome prediction through postoperative follow-up integration.

### 4.2. Facial and Emotional Recognition for Pain Assessment in Non-Communicative Patients

AI-based facial recognition systems have emerged as promising tools for objective pain evaluation in patients unable to self-report, such as neonates, sedated ICU patients, or individuals with cognitive impairment. These systems leverage computer vision and affective computing to detect micro-expressions, autonomic changes, and behavioral cues correlated with pain states. While none of the current articles focused explicitly on this application, the foundational technologies—such as convolutional neural networks, deep learning, and pattern recognition—were extensively discussed in the context of clinical decision-making and patient monitoring. For instance, the work by Wang et al. [18] on hybrid AI models for depth of anesthesia (DoA) prediction illustrates how multimodal input (e.g., EEG signals and infusion data) can be processed using attention-based neural architectures to produce accurate clinical inferences. Similar architectures are being used in current research to analyze facial video data and detect nociceptive responses. In anesthesiology, integrating these models with intraoperative monitoring systems could facilitate real-time pain detection, especially during light anesthesia or sedation, where traditional monitoring may fail to capture nociceptive distress. Such systems also hold promise in postoperative settings where sedation, language barriers, or neurological deficits impair patient communication.

## 5. Regional Anesthesia and Ultrasonography

### 5.1. Computer Vision Assistance for Anatomical Structure Identification

The integration of computer vision algorithms with ultrasonography is transforming regional anesthesia practice by enhancing image interpretation and anatomical landmark recognition. Traditionally, ultrasound-guided regional anesthesia requires a high degree of manual skill and experience to identify target nerves, fascial planes, and adjacent critical structures. AI, particularly deep learning methods such as convolutional neural networks, has demonstrated the capacity to interpret ultrasound images with accuracy comparable to expert clinicians [15]. Kong et al. [12] reviewed the current and emerging applications of AI in obstetric anesthesia and emphasized the growing use of computer vision in identifying neuraxial anatomy for epidural and spinal procedures. These systems assist in automated segmentation of the vertebral column, ligamentum flavum, and epidural space, significantly improving the success rate of neuraxial blocks in challenging patients such as those with obesity or scoliosis. Furthermore, explainable AI models are increasingly applied to enhance clinician trust by providing interpretable outputs during ultrasound-assisted procedures. This is particularly relevant in educational settings, where AI-driven platforms can be used for training anesthesiology residents in real-time ultrasound interpretation, improving both safety and procedural consistency. AI-enhanced systems have demonstrated the ability to accurately identify spinal landmarks, facilitating needle insertion in technically challenging patients, such as those with obesity or abnormal anatomy. In a prospective cohort study, Chan et al. [20] developed and validated an automated ultrasound-guided spinal landmark identification program for spinal anesthesia in obese parturients, achieving a 79.1% first-attempt success rate (95% CI: 65.0–89.5%) and strong correlation (r = 0.915) between algorithm-predicted and clinician-measured needle depth. These technologies, which incorporate graphical user interfaces and real-time image interpretation, not only improve procedural efficiency but also reduce complications associated with multiple puncture attempts.

### 5.2. Needle Navigation and Automated Positioning

Another promising frontier is the development of AI-assisted systems for automated needle guidance. These technologies integrate computer vision with robotic assistance or tracking algorithms to facilitate real-time needle trajectory visualization and adjustment. They aim to reduce the risk of inadvertent vascular or nerve injury, optimize needle-to-target path planning, and increase procedural efficiency. While no studies among the reviewed corpus reported clinical trials of robotic needle navigation, Kong et al. [12] discussed prototype systems capable of tracking needle position relative to ultrasonographic landmarks using AI-enhanced feedback mechanisms. These systems use either image registration or pattern recognition to map anatomical structures dynamically and align the needle path accordingly, adjusting for patient movement or probe repositioning. This technology holds promise for complex regional techniques, such as paravertebral or deep plexus blocks, where visualization and precision are critical. In future implementations, closed-loop systems integrating ultrasound feedback, needle actuation, and AI decision-making could enable semi-autonomous regional anesthesia, especially in high-volume or resource-limited settings [21]. Despite their promise, current limitations include high system costs, the need for real-time image processing at high resolution, and the challenge of generalizing across diverse patient anatomies. Nonetheless, ongoing improvements in AI model training, image quality, and hardware integration suggest these technologies may become part of standard practice in the near future.

## 6. AI in Monitoring the Depth of Anesthesia

AI techniques, particularly those leveraging EEG signal analysis, have shown considerable promise in improving the accuracy and granularity of perioperative monitoring. Gu et al. [22] developed a method combining multiple EEG-derived features—permutation entropy, spectral edge frequency (SEF95), BetaRatio, and SynchFastSlow—with an artificial neural network (ANN) to classify anesthetic depth across four states: awake, light, general, and deep anesthesia. In a cohort of 16 patients under propofol anesthesia, the ANN model achieved a classification accuracy of 79.1%, with strong sensitivity for detecting awake (86.4%), light (73.6%), and general anesthesia (84.4%). The ANN output demonstrated high correlation with the bispectral index (r = 0.892; *p* < 0.001) and outperformed support vector machines in precision and reliability. These results highlight the feasibility of AI-based EEG monitoring systems as advanced tools for real-time depth of anesthesia assessment, offering potential advantages in individualizing anesthetic management and reducing the risks of under- or over-sedation. Jiang et al. [23] proposed a novel ANN model that integrates EEG-based sample entropy (SampEn) with expert anesthesiologist assessments. EEG signals from 64 surgical patients were preprocessed using multivariate empirical mode decomposition and analyzed for SampEn values, which were then used to train and validate the ANN model against expert-assigned consciousness scores. The ANN output demonstrated a stronger correlation with expert evaluations (r = 0.73 ± 0.17) than with bispectral index values (r = 0.62 ± 0.19) and achieved a higher area under the ROC curve (AUC = 0.953 ± 0.07). These results highlight the ANN model’s potential to provide a clinically meaningful and physiologically grounded DoA indicator that may surpass commercially available systems by aligning more closely with anesthesiologist judgment.

## 7. AI in Sedation for Endoscopy Procedures

**Figure 1:**
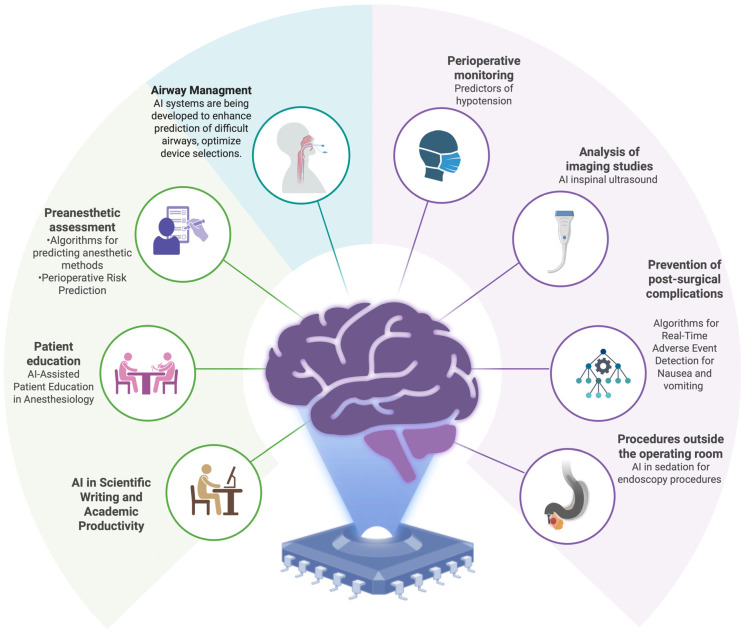
Clinical and Operational Domains of Artificial Intelligence Applications in Anesthesiology.

The application of AI in gastrointestinal endoscopy has extended beyond diagnostic augmentation to support anesthesia quality control in procedural sedation. In a randomized controlled trial, Xu et al. [24] investigated the ENDOANGEL system, a deep learning–based tool that provides real-time procedural cues—such as anatomical landmarks, scope timing, and movement alerts—to anesthetists during sedative endoscopy. By guiding the timing of anesthetic administration, ENDOANGEL enables more precise sedation management, improving patient safety and clinician performance. Patients in the AI-assisted group experienced significantly shorter emergence and recovery times, higher satisfaction levels, and a lower incidence of intraoperative adverse events compared to the control group, underscoring the value of AI-driven procedural awareness in sedation practices. The study enrolled 154 patients who underwent sedative gastrointestinal endoscopy and were randomized into either a control group or a computer-aided diagnosis group using ENDOANGEL. Primary endpoints included emergence time, recovery time, and patient satisfaction, while secondary metrics assessed anesthetic doses, Ramsay sedation scores, and adverse event rates. The computer-aided diagnosis group demonstrated significantly improved outcomes: emergence time (2.73 ± 2.16 vs. 4.73 ± 3.34 min, *p* < 0.01), recovery time (4.01 ± 2.40 vs. 6.55 ± 3.80 min, *p* < 0.01), and patient satisfaction (4.54 ± 0.50 vs. 4.04 ± 0.49, *p* < 0.01). Intraoperative adverse events were also notably reduced (18.4% vs. 34.6%, *p* = 0.023) [24]. These findings suggest that AI-enhanced feedback systems can optimize anesthetic delivery and monitoring in non-operating room anesthesia settings, contributing to more effective and patient-centered care (Figure 1).

## 8. Artificial Intelligence in Anesthesiology Research and Education

Anesthesiology, a specialty requiring high precision and critical decision-making, has historically embraced technological innovations. In recent years, the advent of AI has begun to transform research methodologies and educational paradigms within anesthesiology. From automated literature review and clinical training simulators to support in scientific writing, AI offers unprecedented opportunities to enhance efficiency, accuracy, and scholarly output. However, these advances must be approached with critical oversight, particularly given the patient safety imperatives inherent to anesthetic practice.

### Intelligent Simulators for Anesthesiology Training

The integration of AI into anesthesiology education has introduced innovative strategies for enhancing procedural training, particularly in ultrasound-guided regional anesthesia. Cai et al. [16] conducted a simulation-based randomized study evaluating the impact of an AI-assisted nerve identification system based on convolutional neural networks during training for ultrasound-guided popliteal sciatic nerve blocks. Forty anesthesiology residents were randomized into a traditional teaching group or an AI-assisted group. Residents in the AI group demonstrated a significantly lower incidence of paresthesia during puncture (4.12% vs. 14.06%, *p* < 0.001) and during injection (2.25% vs. 6.64%, *p* = 0.025), as well as higher self-assessment scores (7.53 ± 1.62 vs. 6.49 ± 1.85, *p* < 0.001) and higher post-training performance on the Assessment Checklist for Ultrasound-Guided Regional Anesthesia (32 ± 3.8 vs. 29.4 ± 3.9, *p* = 0.001). Additionally, Kitapcioglu et al. [25] conducted a randomized controlled trial comparing AI-supported voice command interfaces to traditional VR controllers during Advanced Life Support (ALS) training. Although both groups exhibited improvements in knowledge from pre- to post-training, the VR controller group achieved significantly higher examination scores than the voice command group (mean 80.47 vs. 66.70, *p* = 0.005). Notably, while voice command systems enhanced accessibility, technical limitations such as recognition latency and speech errors contributed to reduced performance. This highlights the need for further refinement of AI voice recognition before widespread adoption in critical clinical training contexts. Similarly, Katz et al. [26] investigated the effectiveness of a voice-based VR team leader refresher module compared to traditional high-fidelity simulation for ACLS skills among anesthesiology residents. Although technical performance scores were significantly higher in the high-fidelity simulation group (median 72.7%) compared to the VR group (median 47.0%, *p* < 0.001), both modalities were rated similarly in terms of learner satisfaction and non-technical skills like communication and decision-making. Importantly, VR sessions were substantially more cost-effective and less physically demanding for instructors, suggesting that VR training could play a crucial role in scalable, resource-efficient education strategies in anesthesiology [26]. The studies collectively emphasize that while VR and AI-driven training modules offer promising alternatives or adjuncts to traditional simulation, current limitations—particularly in real-time accuracy and depth of feedback—need to be addressed to fully match the educational quality of high-fidelity simulation. Voice command interfaces, although intuitive and accessible, require enhanced natural language processing capabilities to avoid recognition errors that could impair learner performance and confidence, as seen in both studies. Nonetheless, the scalability, reduced cost, and lower instructor workload offered by VR platforms support their gradual integration into anesthesiology curricula, particularly for refresher training and preliminary skill acquisition. Given that anesthesiology demands rapid, accurate responses under pressure, intelligent simulators represent an invaluable tool for fostering critical thinking and resilience among trainees, ultimately contributing to improved patient outcomes.

## 9. AI in Scientific Writing and Academic Productivity

Systematic literature reviews traditionally require considerable time and cognitive resources. AI-driven platforms such as PubTator 3.0 have revolutionized this process by employing sophisticated natural language processing algorithms to automatically annotate biomedical literature, identify key entities, and establish semantic relationships [27]. PubTator 3.0 has demonstrated a high annotation accuracy (>90% for gene and disease entity recognition) and significantly accelerated literature mining workflows, allowing researchers to rapidly synthesize evolving evidence bases. In anesthesiology, this facilitates the detection of emerging trends such as novel regional anesthesia techniques or new perioperative risk stratification models. In parallel, a study assessing the use of large language models like ChatGPT in scientific writing found that while these tools can expedite manuscript drafting by approximately 30%, they carry a notable risk: up to 12% of generated references were either hallucinated or inaccurately cited [28]. Thus, while AI offers substantial efficiency gains, human oversight remains indispensable to ensure the scientific accuracy and integrity required for evidence-based anesthetic practice. Recent evaluations, such as the study by Granjeiro et al. [29], have systematically assessed AI tools like Elicit, Perplexity, Consensus, ChatGPT, and Grammarly, demonstrating their potential to optimize key stages of the research process, including literature review, synthesis of information, and improvement of linguistic quality. Importantly, Granjeiro et al. [29] emphasized the ethical challenges associated with AI integration into scientific writing. Standardization of narrative style, erosion of individual scholarly voice, potential for undetected plagiarism, and ambiguities around authorship accountability represent pressing issues. Ethical frameworks, such as those recommended by SciELO and Brazilian academic institutions, advocate for mandatory disclosure of AI tool usage in scientific manuscripts and promote human centrality and transparency throughout the research communication process. On the other hand, Aljamaan et al. [28] introduced the Reference Hallucination Score (RHS) as a novel metric to systematically evaluate the authenticity of citations produced by medical AI chatbots, addressing a major gap in the assessment of AI’s academic contributions. The study challenged six widely used AI chatbots (ChatGPT 3.5, Bing, Perplexity, Elicit, SciSpace, and Google Gemini) with standardized medical prompts. Each chatbot was tasked to generate 10 references per prompt. The RHS was constructed by evaluating seven key bibliographic elements: title, journal name, authors’ names, publication date, DOI, web link, and relevance to the prompt keywords. Hallucination was quantified when references contained fabricated, erroneous, or irrelevant information, with higher RHS values indicating greater levels of hallucination. Findings revealed significant disparities among chatbots. Google Gemini failed to generate references for any prompt. In contrast, ChatGPT 3.5 and Microsoft Copilot produced the highest hallucination scores (RHS = 11), indicating a critical risk of reference fabrication. Perplexity showed moderate hallucination (RHS = 7), while Elicit and SciSpace demonstrated the lowest hallucination rates (RHS = 1), closely aligning their outputs with verified bibliographic data. Specifically, hallucination was most frequent in terms of reference relevance (61.6% error rate). The study emphasized that while chatbots like ChatGPT and Microsoft Copilot may generate fluent and convincing bibliographies, a substantial portion of their references are either fabricated or incorrectly attributed, highlighting a serious threat to scientific rigor if such outputs are used uncritically in medical research. In the context of anesthesiology research, where precision, originality, and ethical rigor are fundamental, these insights are crucial. AI tools should be embraced as strategic allies that augment researchers’ capabilities—enhancing literature analysis, accelerating the drafting process, and refining language quality—but always under stringent human validation to preserve the scientific integrity and credibility of the specialty.

## 10. Challenges, Limitations, and Ethical Considerations

**Figure 2:**
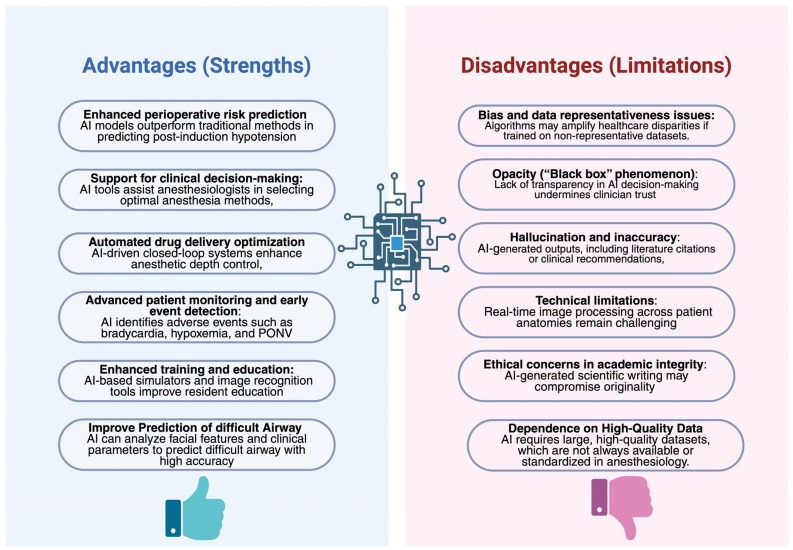
Current Benefits and Limitations of Artificial Intelligence in Anesthesiology.

The rapid advancement of AI technologies has unlocked unprecedented opportunities in anesthesiology, including perioperative monitoring, automated drug delivery systems, and educational innovations. Nevertheless, the widespread adoption of AI is accompanied by substantial technical, clinical, and ethical challenges that necessitate careful scrutiny. Although models such as GPT-4 have demonstrated promising clinical reasoning abilities by achieving passing scores on the American Board of Anesthesiology examinations [30], important limitations persist. These include variability in performance across subspecialties, the phenomenon of information “hallucinations,” and deficiencies in real-time prioritization and decision-making—factors that critically undermine trust in AI-based clinical applications. A major risk associated with AI deployment in anesthesiology relates to the amplification of biases embedded within training datasets. As highlighted by BaHammam [31], AI algorithms may inadvertently perpetuate disparities in healthcare delivery, compromising patient safety if predictive models are trained on unrepresentative populations. Moreover, the emergence of “paper mills,” where AI is used to generate fraudulent scientific articles, poses a significant threat to the integrity of biomedical literature. The opacity of AI models—the so-called “black box” phenomenon—further limits result interpretability, impeding the validation of AI recommendations in high-stakes anesthetic practice [32]. Authorship and attribution controversies constitute another crucial ethical dimension. Recent analyses, including those by Khalifa and Albadawy [33], assert that AI cannot be recognized as an author, as it lacks the capacity for intellectual responsibility. Furthermore, AI-generated content often fails to meet originality standards and may not qualify for copyright protection, complicating its use in scientific publications, clinical guidelines, or research protocols. Non-transparent or inappropriate use of AI in academic writing could undermine a researcher’s credibility and distort the evidence base guiding anesthetic practice. Finally, technical challenges surrounding data security and patient confidentiality are particularly salient. The vast amounts of sensitive information processed by AI tools for perioperative risk prediction and real-time monitoring demand robust cybersecurity frameworks [34]. As outlined by Paiste et al. [35], the lack of standardization across predictive models and the risk of erroneous outputs in critical care settings remain serious concerns. Therefore, the future integration of AI into anesthesiology must be grounded in medical ethics, algorithmic transparency, dedicated clinician training, and continuous human oversight to ensure that these technologies enhance rather than compromise patient safety and the quality of anesthetic care (Figure 2).

The deployment of AI-based systems in anesthesiology—especially those supporting or executing autonomous decisions—demands an ethical framework tailored to the unique time-critical and high-stakes nature of the perioperative environment. While AI can enhance pattern recognition and predictive accuracy, it cannot assume moral responsibility for decisions affecting patient safety. This is particularly relevant in scenarios involving closed-loop anesthesia delivery systems, predictive hemodynamic monitoring, and airway management, where errors of omission or over-reliance on AI suggestions could have immediate consequences.

Ethically, integration of AI into anesthetic workflows must also preserve the principle of proportionality, wherein the benefits of automation do not compromise individualized patient care. For example, while closed-loop systems can titrate anesthetics with high precision, anesthesiologists remain essential for interpreting situational variables such as surgical stimulation, patient comorbidities, and intraoperative events not anticipated by the algorithm.

Furthermore, the responsibility for adverse outcomes arising from AI-assisted decisions must remain clearly delineated. Current legal frameworks do not confer agency or accountability upon AI systems; thus, the final responsibility invariably resides with the human clinician. This reinforces the need for robust validation, clear audit trails, and continued education of anesthesia professionals regarding the capabilities and limitations of AI tools.

The comparative synthesis of AI applications presented in Table 1 and Table 2 highlights the heterogeneity in validation strategies, performance, and generalizability.

While some tools, such as HPI systems or chatbot education, have undergone early clinical trials, others remain in preliminary or simulation-based phases.

## 11. Conclusions

The progressive integration of artificial intelligence into anesthesiology has opened promising avenues for optimizing care across the preoperative, intraoperative, and postoperative phases. However, technological advancements alone do not inherently guarantee a comprehensive improvement in anesthetic practice. Rather, this evolving landscape necessitates a critical and ethically grounded integration in which AI functions as a complementary tool, one that enhances but does not replace the contextual knowledge, technical expertise, and clinical intuition of the anesthesiologist. The concept of hybrid decision-making embodies a mature, interdisciplinary vision for the future of anesthesiology characterized by a dynamic interplay between intelligent systems and highly trained professionals. While AI offers superior analytical capacity in processing complex, high-volume data, it is the anesthesiologist’s irreplaceable role in interpreting clinical ambiguity, exercising real-time judgment, and maintaining the human dimension of care that ensures patient safety and individualized outcomes. Within this virtuous balance, AI can augment the safety, precision, and efficiency of anesthetic management, provided its implementation is guided by principles of algorithmic transparency, rigorous clinical validation, and continuous human oversight. Such a paradigm reinforces the anesthesiologist’s role as a leader in perioperative care, advancing a model of medicine that is not only technologically empowered but also ethically grounded and patient-centered. The anesthetic decision-making of the future will thus emerge as a synthesis of algorithmic reasoning and medical consciousness—driven by innovation, but governed by clinical expertise and prudent judgment.

## Figures and Tables

**Table 1:** Comparative Summary of Key Clinical Studies on AI in Anesthesiology.

Author (Year)	AI Application	Study Design	Sample Size	Key Results	Limitations/Generalizability
Chen et al. (2025) [8]	Post-induction hypotension prediction	Retrospective analysis	5406	AUROC = 0.74 (logistic regression)	Superior to complex models; needs external validation
Çelik et al. (2025) [9]	AI-guided anesthetic choice	Observational comparison	Not stated	AI-human concordance up to 85.7%	Model variability; lacks real-time clinical testing
Yahagi et al. (2024) [10]	Chatbot-assisted patient education	Randomized controlled trial (RCT)	Not stated	Significant anxiety reduction over time (*p* = 0.015)	Content reliability not guaranteed; time-sensitive effects
Shimada et al. (2024) [11]	HPI monitoring for intraoperative hypotension	Meta-analysis of small RCTs	Varies	Trend toward lower hypotension burden	Limited by small trials; moderate heterogeneity
Wijnberge et al. (2020) [17]	ML-based early warning system (HPI)	Randomized controlled trial	60	↓ TWA hypotension (0.10 vs. 0.44 mmHg, *p* = 0.001)	Single-center; requires broader validation
Xu et al. (2022) [24]	AI-assisted sedation monitoring (ENDOANGEL)	Randomized controlled trial	154	↓ emergence/recovery time; ↑ satisfaction (*p* < 0.01)	Generalizability beyond GI endoscopy unconfirmed

**Table 2:** Systematic Comparison of AI Applications in Anesthesiology by Domain.

Application Domain	AI Techniques Used	Validation Type	Performance Metrics	Clinical Barriers
Clinical Records	NLP, text mining	Implementation studies	Improved documentation and extraction accuracy	Low adoption rate, data heterogeneity
Medical Imaging & Ultrasound	CNNs, deep learning, computer vision	Prospective cohorts	Landmark ID success up to 79.1% (Chan et al. [20])	Variability in anatomy; image quality issues
Airway Management	ML classifiers, facial image analysis	Accuracy-based validations	>85% prediction accuracy for difficult airway	Black-box limitations; operator trust
Hemodynamic Monitoring	ML, early warning systems (HPI)	RCTs, meta-analyses	↓ TWA hypotension, ↑ prediction accuracy	Alert fatigue; limited to specific procedures
TIVA/TCI Automation	Hybrid neural networks (LSTM, Transformer)	Model performance (MSE)	MSE = 0.0062 (Wang et al. [18])	Need for real-time pharmacodynamic integration
Patient Education	LLM-based chatbots (e.g., ChatGPT)	Randomized controlled trial	Improved engagement, anxiety modulation (*p* = 0.015)	Reference hallucination; inconsistent output
Procedural Sedation	Deep learning (ENDOANGEL)	Randomized controlled trial	↓ adverse events, ↓ recovery time	Validation limited to endoscopy
Education & Simulation	AI-guided simulators, voice recognition	Simulation-based RCTs	↑ procedural safety; ↓ paresthesia in block placement	Voice recognition errors; cost of simulation
Analgesia & Pain Prediction	Penalized regression, ensemble learning	Model development and internal validation	>80% accuracy for PONV prediction	Sparse data on chronic pain; external validation needed
Depth of Anesthesia Monitoring	ANN + EEG entropy/Spectral analysis	Comparison with BIS and expert assessment	AUC up to 0.95; better correlation than BIS	Noise sensitivity; lack of standardization

## Data Availability

No new data were created or analyzed in this study. Data sharing is not applicable to this article.

## Abbreviations

AI	Artificial Intelligence
ML	Machine Learning
DL	Deep Learning
CNN(s)	Convolutional Neural Network(s)
EEG	Electroencephalogram
TIVA	Total Intravenous Anesthesia
TCI	Target-Controlled Infusion
ASA	American Society of Anesthesiologists
POCUS	Point-of-Care Ultrasound
HPI	Hypotension Prediction Index
AUROC	Area Under the Receiver Operating Characteristic
LASSO	Least Absolute Shrinkage and Selection Operator
ANN	Artificial Neural Network
SEF95	Spectral Edge Frequency 95%
ROC	Receiver Operating Characteristic
AUC	Area Under the Curve
DoA	Depth of Anesthesia
SampEn	Sample Entropy
VR	Virtual Reality
ALS	Advanced Life Support
ACLS	Advanced Cardiac Life Support
RHS	Reference Hallucination Score
GPT-4	Generative Pre-trained Transformer 4
DOI	Digital Object Identifier
ICU	Intensive Care Unit
